
JOURNAL INFORMATION
==============================
NLM Title Abbreviation: Behav Neurol
Journal ID: Behav Neurol
NLM Journal ID: 8914585
Journal ID: BN

Behavioural Neurology

ISSN: 0953-4180
EISSN: 1875-8584
Publisher: Wiley

ARTICLE INFORMATION
==============================
PMCID: PMC5471531
PMID: 17148832
DOI: 10.1155/2006/164397
Article ID: 164397
Article version: 1
Subjects: Editorial

Manipulating Brains

Walsh Vincent 1
Desmond John E. 2
Pascual-Leone Alvaro 3
1 Institute of Cogntive Neuroscience and Department of Psychology University College London UK
2 Department of Neurology Johns Hopkins University Baltimore MD USA
3 Center for Noninvasive Brain Stimulation Harvard Medical School and Beth Israel Deaconess Medical Center Boston MA USA
Print publication date: 2006
Electronic publication date: 2006 Nov 27
Volume: 17
Issue: 3-4
First page: 131
Last page: 134
Received 2006 Nov 21; Accepted 2006 Nov 21
Copyright: Copyright © 2006 Hindawi Publishing Corporation and the authors.
Copyright year: 2006
License: This is an open access article distributed under the Creative Commons Attribution License, which permits unrestricted use, distribution, and reproduction in any medium, provided the original work is properly cited.
License URL: https://creativecommons.org/licenses/by/3.0/

==============================
Behavioural Neurology 17 (2006) 131–134
IOS Press

Editorial

### Manipulating brains

Vincent Walsh[a], John E. Desmond[b] and Alvaro Pascual-Leone[c]
[a]*Institute of Cogntive Neuroscience and Department of Psychology, University College London, UK*
[b]*Department of Neurology, Johns Hopkins University, Baltimore, MD, USA*
[c]*Center for Noninvasive Brain Stimulation, Harvard Medical School and Beth Israel Deaconess Medical Center, Boston, MA, USA*

### 1. Introduction

The desire to manipulate brain function is not new. In manipulating brain function the goal is to manipulate behaviour and we have been manipulating behaviour for the whole of our evolution as social animals. Primates, such as ourselves, living in social groups carry out mind manipulation with astonishing speed and accuracy throughout the day. We are all a little friendlier, some unknowingly, others less so, when we want something. We manipulate each other's wishes, patience, value judgments and beliefs. We are so good at it that we can do it en masse: The smell of bread in the supermarket entrance, the classy music in a clothes store, and the promise of instant health on a snack bar wrapper are all ways of manipulating behaviour and thus the nervous system. Some are real masters of this sort of manipulation and succeed in leading entire nations into huge successes or horrible abominations. For most of us, basic techniques on how to master such manipulations of others to maximize personal success can be learned from the like of Dale Carnegie and his 1936 classic *"How to Win Friends and Influence People"* [1]. So, if we can manipulate the behaviour of individuals and populations so subtly and successfully, why do we struggle in our attempts to help stroke victims walk and talk? If it is so easy to make people want things, why is it so hard to make them learn things? And if we can manipulate minds so naturally, why can we not construct machines, therapies and drugs to do it too?

The answer is twofold and encapsulates the problems, which the papers in this issue try to address. The first part of the answer is that the most successful machine for mind manipulation is another mind: our brains are optimized for the sending and decoding of social signals by millions of years of evolution. The second part of the answer is that the evolution of minds for mutual mind manipulation has proceeded entirely on the basis of the outcome of attempts at manipulation – for one mind to manipulate another requires no knowledge of the mechanisms operating in that mind. Our struggle to comprehend these mechanisms by understanding the biological basis of the mind leaves us with at least three problems: How to physically manipulate brain structure and function; how to achieve long-term effects of our interventions; and how to understand the integrated nature of brain function while having the goal of achieving behaviourally local effects.

The premise behind this issue of Behavioural Neurology is that it is both possible and in a clinical context desirable to influence brain function and that it can serve to improve behaviour and promote recovery of function. As brain imaging techniques improve, scientist are becoming increasingly likely to eventually predict, by examining a scan of a person's brain, whether he or she will tend to depression or violence, or whether he or she has talents in certain areas. We are likely to learn not only about the brain areas involved in lying, working responsibly, and acquiring new skills with far greater precision than we know now, but also how to change them to enhance or suppress their function and hence manipulate behaviour. Neural implants may within a few years be able to increase in-

ISSN 0953-4180/06/$17.00 © 2006 – IOS Press and the authors. All rights reserved telligence, speed up learning, modify emotional states, or enhance motor functions. Drug companies are hunting for molecules to alleviate brain-related symptoms, from paralysis to shyness. We are learning more and more about how the brain changes and generates behaviour and at the same time about how to manipulate the brain. Indeed, if we accept that the brain changes with each experience, parents and educators have been modulating young persons' brains for centuries with ever more refined techniques. Education is a way of modifying brain activity, hence changing it and influencing behaviour. Perhaps neurotechnology offers the advantage of being more precise, more controlled, more specific in its actions and thus less prone to undesirable side-effects. However, we are not visiting new territories in the goal of brain manipulation and we should perhaps arm ourselves with some historical awareness of how others have approached it.

### 2. Hard lessons of history

Two strands of mind manipulation in history – Mesmerism and phrenology – receive fairly hard knocks in many textbooks and popular books. Mesmer's interventions were based on faith in animal magnetism. Mesmer, like many others, correctly noted that people's behaviour could be influenced by their mental state. However, there was no sense in which knowledge about mechanisms of brain function played a part in his early form of mind manipulation, and the French Royal Commission was correct when it reported in 1784 that in Mesmer's magnetising "too many things are seen at once for any one thing to be seen well". We should dampen feelings of superiority, however, and ask whether the conclusion of the Commission would not be an inappropriate referee's comment on brain imaging experiments that observe thirty activations but confidently discuss only two of them; or on magnetic stimulation experiments that report functional improvements that cannot be explained on the basis of known anatomical connections? Phrenologists, on the other hand, surely deserve a better press. It is true that as the movement gathered pace the number of areas and the descriptions of functions became dizzying. Nevertheless, it is worth remembering that Gall can be credited with being the first scientific monist (a belief for which he suffered social and professional consequences), to focus scientific attention on the grey matter of the brain, and, though the link between skull shape and brain function turned out to be wrong, with describing the first systematic conceptualisation of localisation of function. A resurrected Gall would likely soon be up to speed if his notions of bumps were replaced with those of blood oxygenation levels.

One of the first attempts at brain stimulation was in 1755 when Charles le Roy applied electrodes to the forehead of a young blind man in order to stimulate his eyes and hopefully restore vision (Fig. 1). The patient, a 21 year-old Englishman, perceived vivid phosphenes over several days of treatment. He remained blind. With the emerging knowledge that nervous tissue responded to electricity the idea of exposing our bodies and brains to electrical and magnetic stimulation soon became a source of popular and professional hope. But simply getting to the brain proved to be a task in itself. Towards the end of the $19^{th}$ century physiologists and physicians began to realise that large magnetic fields may be able to generate electrical fields in the brain and therefore stimulate brain tissue. The two main stumbling blocks were the need for large currents as well as the rapid alternation of current. D'Arsonval, for example, wrote that "an alternating magnetic field with an intensity of 110 V, 30 amperes and a frequency of 42 cycles per second, gives rise to, when one places the head into the coil, phosphenes and vertigo, and in some persons, syncope" (Fig. 2). These early attempts, however, did not reach the brain, they merely stimulated the retina, and it was not until 1985 that it became possible to use magnetic fields to stimulate the brain painlessly. In the interim, magnetic fields acquired the name of "Odic Forces" and the New York press was able to announce in 1907, that "Science finds a new fountain of youth for tired lazy and nervous people". The power of these forces even made it into the classroom and the nursery (Fig. 3) as parents succumbed to the explanations given for, in the words of another news headline from 1912, "Why children are made brighter by electrifying them".

Again we should beware of any nascent feelings of superiority. These early attempts were finding a way towards answering the question of how to physically manipulate brain structure and function. By the 1960s it was routine to stimulate animals' brains with implanted electrodes, and good control over some basic functions such as hunger, thirst and fear could be exercised. It became clear that some functions could be localised and that dramatic effects could be elicited, with a particularly famous and striking example being that of Jose Maria Delgado's dramatic demonstrations of control over the motivation of animals (Fig. 4). However, this raised – and for some continues to raise – ethical questions about the cost/benefit analysis of brain manipulation. Jose Maria Delgado eventually proposed the creation of a ‘psychocivilized society’ [4] in which guided brain stimulation could be used to modify ‘appropriately’ each individual’s character and thus make it ‘best suited to the general good’. Such suggestions echo dangerously of Huxley’s “Brave New World” [7] and raise ethical concerns as to the definition of ‘appropriate character’ and ‘general good’.

### 3. Modern mind manipulation approaches

Genes certainly affect brain anatomy and function. Indeed, even if a genetic inheritance is not readily obvious from epidemiologic or twin studies, predisposing genes can still be critically responsible for illnesses and character traits. In that sense, manipulating the genetic make-up of a person may well change brain function and ultimately behaviour. However, to think that there is a one-to-one, precise mapping between genetic code and behaviour is simply naïve. A person’s genetic make-up certainly has something important to do with his/her subsequent behaviour, but genes exert their effects through the brain so that if you want to predict and control a person’s behaviour, the brain itself is the place to start. Moreover, the same behaviour can be the consequence of different patterns of brain activity and hence different brain anatomies and genetic makeups. Therefore, changing genes will likely not allow us to modify behaviour in a controlled and predictable way, while more immediate brain manipulations, such as brain stimulation, might.

Pharmacological interventions are similarly less likely to allow controlled manipulations of brain activity and behaviour than guided brain stimulation. Precise delivery of neuropharmacologic agents may allow controlled behavioural manipulations, but that might be easiest to achieve by using brain stimulation techniques to prime and thus define the targets of medications.

Transcranial stimulation offers a non-invasive methodology to explore such potential. Noninvasive brain stimulation provides a valuable tool for (1) interventional neurophysiology applications, modulating brain activity in a specific, distributed, cortico-subcortical network so as to induced controlled and controllable manipulations in behavior, as well as for (2) focal neuropharmacology delivery, through the release of neurotransmitters in specific networks [13] and the induction of focal gene expression, that may yield specific behavioral impact [8]. Furthermore, transcranial stimulation studies can serve as a proof of principle to inform future more invasive techniques involving implantation of electrodes and chronic brain stimulation [6,9]. For example, transcranial magnetic stimulation, particularly when applied repetitively in short trains, can modulate the excitability of the targeted brain region beyond the duration of the stimulation train itself [10] and exert distant effects along functional neural networks [11]. Such a modulation of activity in a distributed neural network can result in a functional gain, improve the performance in specific tasks, and pose a therapeutic potential in neurology, psychiatry or rehabilitation as well as a behavioural advantage for normal subjects under certain circumstances.

### 4. So where are we now?

We have better equipment than le Roy or d’Arsonval, know more about the brain than Gall and therefore should have better means to manipulate and guide behaviour and better estimates of long term effects. In the case of magnetic stimulation we have a very good idea of the spatial and temporal specificity of the intervention. We also have good control of whether, in the motor system at least, one is stimulating excitatory or inhibitory pathways. We also know how magnetic stimulation interacts with certain drugs. Consequently magnetic stimulation has been used in a wide variety of proof-of-principle and pilot therapeutic trials for a variety of conditions. Most of the beneficial effects are short lived, however, and a great deal of work remains to be done to test their interaction with behavioural and pharmacological interventions. It now seems clear that 1 Hz stimulation for several minutes [2] or theta burst stimulation for a few tens of seconds [5] can alter brain excitability for the many minutes afterwards. These periods of changed excitability may provide windows of opportunity during which one might recruit previously compromised components of the neural circuits into contributing to behavioural recovery or plasticity. However the brain is not very good at being passive and the effect that one has during magnetic stimulation depends on the state of activation of the brain at the time of that stimulation. This was demonstrated very clearly by Classen et al. [3] who showed that simply practising moving one’s thumb in a given direction for a few minutes changed the direction of a thumb movement subsequently induced by magnetic stimulation. The brain we are stimulating is neither passive nor neutral and this makes the challenge to understand the integrated nature of brain function while having the goal of achieving behaviourally local effects much more difficult. As has been noted before by Robertson et al. [12] even highly structured interventions in the motor system, for example different methods of teaching piano, can have unexpected consequences as a result of the brain's attempts to adapt. The challenge for anyone embarking on neurorehabilitation research is to find the optimal brain state for the behavioural and physiological interventions intended to help the patient. This also becomes an important ethical consideration, and thus, the ethical debate has to be approached openly and early. Certainly, new knowledge is generally desirable and will likely lead to better tools to combat disease, enhance recovery after brain damage, and minimize disability. However, the risk of attempting to redefine "normal" and aim at manipulating behaviour to enhance desired capacities looms. Given such notions, a public debate over the ethical limits to such neuroscientific applications is essential.

Despite the ethical challenges potentially raised by magnetic stimulation, there are some reasons for measured optimism. The first is that the baseline of suffering against which we are struggling: neuropsychiatric diseases, such as stroke, chronic pain, or depression that may be amenable to therapy by brain stimulation, are major causes of death and long term disability in the developed world. The second is that *plasticity is the normal state of the nervous system* [14, p. 164], and thus the brain is continuously learning and adapting based on the consequences of behaviour. Perhaps, then, there may be situations in which it is sufficient to assist a brain region in relearning for brief periods by inhibiting competing regions, facilitating local activity, or suppressing activity to promote change. When we learn to walk, talk and write as children we do so with many periods of short practice. When stroke patients learn to walk, talk and write again they may also expect many long hours of spaced practice. When we consider the small, short-lived physiological effects of magnetic stimulation in comparison to the activation induced by a page of text, a song or the act of brushing one's teeth it becomes conceivable that brief changes in excitation or inhibition may be sufficient to make a single period of training better than the last one and in doing so change the motivation and belief of the patient. This may be sufficient in some cases, but the higher goal is to define principles of brain reorganisation so that recovery of function can be enhanced and we can better understand the neural basis of normal learning. These are high goals indeed and we may in the future find ourselves being viewed in the same historical light as the purveyors of Odic forces. But, those supreme mind manipulators – our own minds – will just not let us stop trying, and as the papers in this issue show, there are signs that the effort is paying off.

### References

[1] D. Carnegie, *How to Win Friends and Influence People*, Simon and Schuster, New York, 1936.

[2] R. Chen, J. Classen, C. Gerloff, P. Celnik et al., Depression of motor cortex excitability by low-frequency transcranial magnetic stimulation, *Neurology* **48** (1997), 1398–1403.

[3] J. Classen, J. Liepert, S.P. Wise, M. Hallett et al., Rapid plasticity of human cortical movement representation induced by practice, *J Neurophysiol* **79** (1998), 1117–1123.

[4] J.M. Delgado, *Physical Control of the Mind: Toward a Psychocivilized Society*, Harper & Row, New York, NY, 1969.

[5] Y.Z. Huang, M.J. Edwards, E. Rounis, K.P. Bhatia et al., Theta burst stimulation of the human motor cortex, *Neuron* **45** (2005), 201–206.

[6] F.C. Hummel and L.G. Cohen, Non-invasive brain stimulation: a new strategy to improve neurorehabilitation after stroke?, *Lancet Neurol* **5** (2006), 708–712.

[7] A. Huxley, *Brave New World*, HarperCollins Publishers, Inc., New York, NY, 1932.

[8] K. Kudo, M. Yamada, K. Takahashi, G. Nishioka et al., Repetitive transcranial magnetic stimulation induces kf-1 expression in the rat brain, *Life Sci* **76** (2005), 2421–2429.

[9] C.G. Mansur, F. Fregni, P.S. Boggio, M. Riberto et al., A sham stimulation-controlled trial of rTMS of the unaffected hemisphere in stroke patients, *Neurology* **64** (2005), 1802–1804.

[10] A. Pascual-Leone, J. Valls-Sole, E.M. Wassermann and M. Hallett, Responses to rapid-rate transcranial magnetic stimulation of the human motor cortex, *Brain* **117** (1994), 847–858.

[11] T. Paus, R. Jech, C.J. Thompson, R. Comeau et al., Transcranial magnetic stimulation during positron emission tomography: a new method for studying connectivity of the human cerebral cortex, *J Neurosci* **17** (1997), 3178–3184.

[12] E.M. Robertson, H. Theoret and A. Pascual-Leone, Studies in cognition: the problems solved and created by transcranial magnetic stimulation, *J Cogn Neurosci* **15** (2003), 948–960.

[13] A.P. Strafella, T. Paus, J. Barrett and A. Dagher, Repetitive transcranial magnetic stimulation of the human prefrontal cortex induces dopamine release in the caudate nucleus, *J Neurosci* **21** (2001), RC157.

[14] V. Walsh and A. Pascual-Leone, *Transcranial Magnetic Stimulation: A Neurochronometrics of Mind*, The MIT Press, Cambridge, Massachusetts, 2003.
